# An NCBP3-Domain Protein Mediates Meiotic Silencing by Unpaired DNA

**DOI:** 10.1534/g3.120.401236

**Published:** 2020-04-14

**Authors:** Erin C. Boone, Hua Xiao, Michael M. Vierling, Logan M. Decker, Victor T. Sy, Rana F. Kennedy, Marilyn A. Bonham, Shannon F. Schmitz, Annie M. John, Thomas M. Hammond, Patrick K. T. Shiu

**Affiliations:** * Division of Biological Sciences, University of Missouri, Columbia, Missouri 65211 and; † School of Biological Sciences, Illinois State University, Normal, Illinois 61790

**Keywords:** cap-binding proteins (CBPs), meiosis, meiotic silencing by unpaired DNA (MSUD), *Neurospora crassa*, RNA interference (RNAi)

## Abstract

In the filamentous fungus *Neurospora crassa*, genes unpaired during meiosis are silenced by a process known as meiotic silencing by unpaired DNA (MSUD). MSUD utilizes common RNA interference (RNAi) proteins, such as Dicer and Argonaute, to target homologous mRNAs for silencing. Previously, we demonstrated that nuclear cap-binding proteins NCBP1 and NCBP2 are involved in MSUD. We report here that SAD-8, a protein similar to human NCBP3, also mediates silencing. Although SAD-8 is not essential for either vegetative or sexual development, it is required for MSUD. SAD-8 localizes predominantly in the nucleus and interacts with both NCBP1 and NCBP2. Similar to NCBP1 and NCBP2, SAD-8 interacts with a component (Argonaute) of the perinuclear meiotic silencing complex (MSC), further implicating the involvement of cap-binding proteins in silencing.

A gene present in an unusual copy number may indicate a virus or transposon on the move. It is not surprising that eukaryotes often maintain genome surveillance mechanisms to combat these repetitive elements. In *Neurospora crassa*, one such defense mechanism is known as meiotic silencing by unpaired DNA (MSUD) (Aramayo and Metzenberg 1996; Shiu *et al.* 2001). In MSUD, a meiotically unpaired gene triggers the silencing of all copies of that gene for the duration of sexual development. MSUD can be thought of as a specialized RNA interference (RNAi) system that protects the genome integrity of an organism during meiosis (Hammond 2017; Torres-Martínez and Ruiz-Vázquez 2017). In our working model, MSUD begins with the detection of an unpaired DNA segment at the homolog pairing stage, with the guidance of the SAD-6 (suppressor of ascus dominance-6) homology search protein (Samarajeewa *et al.* 2014). A single-stranded aberrant RNA (aRNA) is made from the unpaired DNA and exported to the perinuclear region, *i.e.*, the region just around the nucleus. There, the aRNA reaches the meiotic silencing complex (MSC), which contains a host of RNAi-related proteins (Decker *et al.* 2015). One of these silencing factors is the SAD-1 RNA-directed RNA polymerase (RdRP), which converts the aRNA into a double-stranded RNA (dsRNA) (Shiu and Metzenberg 2002). SAD-3, a putative helicase, may assist SAD-1 in dsRNA synthesis by increasing its processivity on RNA templates (Hammond *et al.* 2011a). The dsRNA is then processed into small interfering RNAs (siRNAs) by the DCL-1 Dicer-like protein (Alexander *et al.* 2008; Hammond *et al.* 2013a). With the help of the QIP (QDE-2-interacting protein) exonuclease, the siRNA duplexes are made into single strands, which subsequently guide the SMS-2 (suppressor of meiotic silencing-2) Argonaute to silence complementary mRNAs (Lee *et al.* 2003; Xiao *et al.* 2010). SAD-2 is a scaffold protein responsible for bringing SAD-1 and others to the perinuclear region (Shiu *et al.* 2006; Decker *et al.* 2015). The precise functions of SAD-4, SAD-5, and SAD-7 are unclear, although the first two are necessary for siRNA biogenesis and the last one may coordinate nuclear and extranuclear silencing events (Hammond *et al.* 2013a, b; Samarajeewa *et al.* 2017).

In addition to the aforementioned silencing components, the nuclear cap-binding complex (CBC) has also been shown to play a role in MSUD (Decker *et al.* 2017). In eukaryotes, precursor mRNAs (pre-mRNAs) undergo several processing events before their translation. One of these modifications is the addition of a 7-methylguanosine to the 5′ end of a nascent transcript. This 5′ cap structure is bound by CBC, which recruits other factors to the transcript and mediates its expression (Gonatopoulos-Pournatzis and Cowling 2014). CBC consists of nuclear cap-binding proteins NCBP1 and NCBP2, also known as CBP80 and CBP20, respectively. This complex is important for transcription, splicing, pre-mRNA 3′ end processing, mRNA stability, RNA export, the pioneer round of translation, nonsense-mediated mRNA decay, and miRNA biogenesis. In mammals, a third nuclear cap-binding protein called NCBP3 has also been characterized (Gebhardt *et al.* 2015, 2019). NCBP3 forms an alternative CBC with NCBP1 and is critical to antiviral defense. In this study, we have shown that an NCBP3-like protein is required for silencing in *Neurospora*.

## Materials and Methods

### Fungal methods and genotypes

Standard fungal techniques were used according to the *Neurospora* protocol guide (http://www.fgsc.net/Neurospora/NeurosporaProtocolGuide.htm). Genotypes of strains used in this study are listed in Table 1. Genetic markers and knockout mutants are originally from the Fungal Genetics Stock Center (FGSC) (McCluskey *et al.* 2010) and the Neurospora Functional Genomics Group (Colot *et al.* 2006). Fungal isolates were cultured on Vogel’s medium (Vogel 1956). Crosses were performed on synthetic crossing medium (Westergaard and Mitchell 1947).

**Table 1:** *Neurospora* strains used in this study

Strain	Genotype
F2-01	*fl A* (FGSC 4317)
F2-29	*rid r*^∆^::*hph*; *fl A*
F2-35	*his-3*^+^::*act*^+^; *fl A*
F2-36	*his-3*^+^::*bml^R^*; *fl A*
F3-23	*rid his-3*^+^::*asm-1*^+^; *fl*; *asm-1*^∆^::*hph A*
F7-14	*fl*; *sad-8*^Δ^::*hph A*
F8-36	*fl*; *yfpn-cbp20*::*nat1*; *yfpc-sad-8*::*hph cbp80*^Δ^::*hph a*
P3-08	Oak Ridge wild type (WT) *a* (FGSC 2490)
P3-25	*mep sad-1*^∆^::*hph a*
P21-10	*rid*; *mus-52*^∆^::*bar yfpn-cbp20*::*nat1*; *yfpc-cbp80*::*hph a*
P21-11	*rid*; *mus-52*^∆^::*bar yfpn-cbp20*::*nat1*; *yfpc-cbp80*::*hph A*
P23-11	*sad-8*^∆^::*hph a*
P23-12	*r*^Δ^::*hph*; *sad-8*^Δ^::*hph a*
P25-68	*rid his-3*; *yfpn-cbp20*::*nat1*; *yfpc-sad-8*::*hph a*
P25-69	*rid*; *yfpn-cbp20*::*nat1*; *yfpc-sad-8*::*hph A*
P25-72	*rid*; *yfpc-sad-8*::*hph*; *yfpn-sms-2*::*hph A*
P26-01	*rid*; *yfpc-sad-8*::*hph*; *yfpn-sms-2*::*hph a*
P26-02	*sad-8*^∆^::*hph a* (FGSC 18314)
P26-03	*sad-8^∆^*::*hph A* (FGSC 18315)
P27-13	*rid nup120-mCherry*::*hph his-3*; *gfp-sad-8*::*hph A*
P27-14	*rid nup120-mCherry*::*hph his-3*; *gfp-sad-8*::*hph a*
P27-15	*rid*; *yfpc-sad-8*::*hph yfpn-cbp80*::*hph A*
P27-16	*rid*; *yfpc-sad-8*::*hph yfpn-cbp80*::*hph a*
P27-17	*rid*; *yfpn-cbp20*::*nat1*; *yfpc-sad-8*::*hph cbp80*^Δ^::*hph A*

Genetic loci are described in the e-Compendium (http://www.bioinformatics.leeds.ac.uk/∼gen6ar/newgenelist/genes/gene_list.htm).

### Knockout library screen and quantitative assay of MSUD suppression

Identification of MSUD suppressors from the *Neurospora* knockout library was as described (Hammond *et al.* 2011a). Quantitative analysis of mutants’ meiotic silencing abilities was conducted in 24-well microplates using an established protocol (Xiao *et al.* 2019).

### Sequence and phylogenetic analyses

Chromosomal location, gene model, and other information for *sad-8* (*ncu01310-t26_1*) are available from FungiDB (Stajich *et al.* 2012). The SAD-8 sequence was used to search the National Center for Biotechnology Information (NCBI)’s conserved domain database (CDD v3.16) for functional domains (Marchler-Bauer *et al.* 2017) and non-redundant protein sequence database (BLASTP v2.8.0+) for homologs (Camacho *et al.* 2009). A neighbor-joining tree for SAD-8-like proteins was constructed using the p-distance method and a bootstrap test of 1000 replicates in MEGA7 v7.0.26 (Kumar *et al.* 2016). Fungal classification was based on the NCBI’s taxonomy database (https://www.ncbi.nlm.nih.gov/taxonomy). A comprehensive fungal tree of life can be seen in the work of Spatafora *et al.* (2017).

### Gene expression analysis

To compare the expression patterns of various silencing genes, *Neurospora* vegetative (SRR080688, SRR081479, SRR081546, and SRR081586) and sexual (SRR957218) RNA-seq datasets were obtained from the European Bioinformatics Institute (EBI)’s European Nucleotide Archive (ENA) (Ellison *et al.* 2011; Samarajeewa *et al.* 2014). These datasets were aligned to predicted *Neurospora* transcripts using Bowtie 2 v2.2.3 (Langmead and Salzberg 2012). Transcript levels, as measured in fragments per kilobase of exon per million mapped reads (FPKM), were calculated using eXpress v1.5.1 (Roberts and Pachter 2013) and compiled in Microsoft Excel v15.4.

### Nucleic acid methods and transformation

Standard molecular techniques were used throughout this work (Sambrook and Russell 2001). Fungal DNA samples were isolated from conidia (asexual spores) (Henderson *et al.* 2005) and vegetative hyphae (Qiagen DNeasy Plant Mini Kit). *Neurospora* transformation by electroporation of conidia was conducted according to Margolin *et al.* (1997). Vectors for fluorescent protein tagging were constructed with the double-joint polymerase chain reaction (DJ-PCR) method (Hammond *et al.* 2011b; Samarajeewa *et al.* 2014), using a Bio-Rad MJ Mini Thermal Cycler. For PCR-based genotype screening and confirmation, the Promega GoTaq Green Master Mix and the Roche Expand Long Range dNTPack were used. DNA sequencing, when necessary, was performed by the University of Missouri (MU) DNA Core. Primers used in strain construction and confirmation are listed in Supplemental Material, Table S1.

### Bimolecular fluorescence complementation (BiFC)

As an *in vivo* protein-protein interaction assay, BiFC is based on the reassembly of the yellow fluorescent protein (YFP) when its nonfluorescing halves are brought within proximity by two interacting proteins (Hu *et al.* 2002; Bardiya *et al.* 2008). BiFC constructs were generated in the manner of Hammond *et al.* (2011b).

### Photography and microscopy

Z-stack pictures of protoperithecia (female structures) were taken using an M205 FA stereomicroscope and a DFC345 FX camera from Leica. For photography of perithecia (fruiting bodies) and asci (spore sacs), an Apple iPhone 5 with a Magnifi photoadapter (Arcturus Labs, Lawrence, KS) and a Vanguard 1231CM microscope were employed. For fluorescent microscopy, Zeiss LSM710 and Olympus BX61 were used. Preparation and visualization of asci were carried out as previously described (Alexander *et al.* 2008; Xiao *et al.* 2010).

### Data availability

Strains are available upon request. The authors state that all data necessary for confirming the conclusions presented in the article are represented fully within the article. Supplemental material available at figshare: https://doi.org/10.25387/g3.12115857.

## Results

### Identification of a semidominant MSUD suppressor

To discover new MSUD players, we have developed a high-throughput reverse genetics screen to isolate silencing mutants from the *Neurospora* knockout library (Hammond *et al.* 2011a; Xiao *et al.* 2019). Using this screening method, we identified two additional strains [FGSC 18314 (*a*) and 18315 (*A*)] that appear to be MSUD-deficient. These strains refer to deletion mutants of *ncu01310*, in opposite mating types. To verify that *ncu01310* is truly important for silencing, we put it through a quantitative MSUD suppression assay.

*Neurospora* typically yields black American football-shaped ascospores (sexual spores). However, if only one *Round spore* copy is present in a cross (*i.e.*, *r*^+^ × *r*^∆^), it will be unpaired and silenced, leading to the production of mainly round ascospores (Shiu *et al.* 2001). This abnormal phenotype can be mitigated if the silencing process is compromised, for example, by having an MSUD gene itself unpaired and silenced (*e.g.*, *sad-1*^+^ × *sad-1*^∆^). This negative feedback scheme is known as “silencing the silencer”. Genes other than *Round spore* have been used as reporting markers for MSUD activity. These include *actin* (*act*^+^), *β-tubulin* (*bml^R^*), and *Ascospore maturation-1* (*asm-1*^+^), whose unpairings result in the production of lollipop asci, wavy asci, and white ascospores, respectively. As seen in Table 2, a deletion mutation of *ncu01310* acts as a semidominant suppressor of MSUD, suggesting that this gene is important for the silencing process. Accordingly, *ncu01310* is referred to as *sad-8* hereafter.

**Table 2:** *sad-8*^Δ^ (*ncu01310*^Δ^) acts as a semidominant suppressor of MSUD

	::*act*^+^ (F2-35)	::*bml^R^* (F2-36)	*asm-1*^Δ^ (F3-23)	*r*^Δ^ (F2-29)
WT (P3-08)	13.4 × 10^3^	0.5 × 10^3^	5.9%	0.0%
*sad-8*^Δ^ (P23-11)	136.3 × 10^3^	19.7 × 10^3^	73.4%	3.2%
*sad-1*^Δ^ (P3-25)	135.9 × 10^3^	353.3 × 10^3^	86.9%	97.6%
	(spores)	(spores)	(black)	(football)

Each MSUD tester (::*act*^+^,::*bml^R^*, *asm-1*^Δ^, or *r*^Δ^) is designed to unpair a reporter gene during meiosis, either by insertion (::) or deletion (^Δ^). In an MSUD-proficient cross, these unpairings result in the reduced production of black American football-shaped ascospores. However, if MSUD is deficient (*e.g.*, when a *sad* gene is unpaired and subjected to self-silencing), the aberrant phenotype can be partially (*e.g.*, tester × *sad-8*^Δ^) or near-fully (*e.g.*, tester × *sad-1*^Δ^) alleviated, depending on the strength of the suppressor.

### SAD-8 is an NCBP3-domain protein

**Figure 1:**
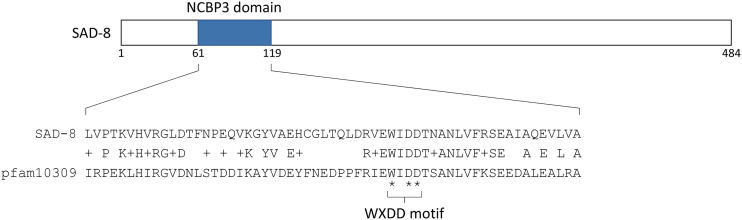
Topography of SAD-8. SAD-8 contains an NCBP3 (pfam10309) domain, which provides a plastic RNA-binding platform. The WXDD motif is a conserved sequence at the center of the RNA-binding groove. Asterisks denote residues important for capped RNA binding in humans (Gebhardt *et al.* 2015).

The *sad-8* gene is located on the right arm of linkage group V. Its translated polypeptide consists of 484 amino acids and has a molecular weight of 54.9 kD. According to the NCBI’s conserved domain database, the N-terminal quarter of SAD-8 contains an NCBP3 (pfam10309) domain, which is a type of RNA recognition motif (RRM) (Figure 1). NCBP3 is a mammalian nuclear cap-binding protein, and it is proposed to form an alternative CBC with NCBP1 (Gebhardt *et al.* 2015, 2019). In humans, this alternative CBC contributes to mRNA export and becomes pivotal under environmental stress, such as during viral infection. The *sad-8* gene is the only open reading frame in the *Neurospora* genome encoding an NCBP3 domain.

**Figure 2:**
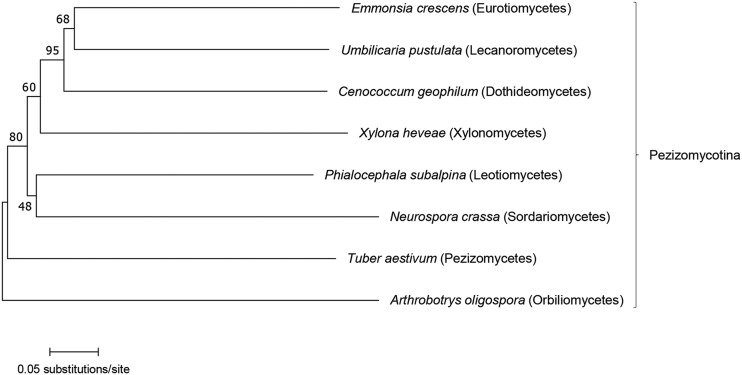
Phylogenetic tree of SAD-8 homologs from the Pezizomycotina subphylum. Numbers next to branches are percentages of bootstrap support. Only one fungal species (in italics) from each class (in parentheses) was included in the tree construction. At the time of the analysis, proteins similar to either *Neurospora* SAD-8 or human NCBP3 were not found in five classes of Pezizomycotina (Arthoniomycetes, Coniocybomycetes, Geoglossomycetes, Laboulbeniomycetes, and Lichinomycetes). GenBank/NCBI Reference Sequence numbers for the proteins analyzed in this study are as follows (from top to bottom): PGH35036.1, SLM34393.1, OCK93975.1, XP_018190555.1, CZR54269.1, XP_961396.1, CUS07653.1, and XP_011123650.1.

As noted by Gebhardt *et al.* (2015), NCBP3-family proteins can be found in the animal and fungal kingdoms. Within Ascomycota (whose members are commonly known as sac fungi), these proteins are seen in all three of its subphyla: Pezizomycotina (*e.g.*, *Aspergillus fumigatus*), Saccharomycotina (*e.g.*, *Candida albicans*), and Taphrinomycotina (*e.g.*, *Schizosaccharomyces pombe*). *Neurospora* belongs to Pezizomycotina, and SAD-8 homologs are represented in several classes of this subphylum (Figure 2).

### Gene expression of SAD-8 during both sexual and asexual cycles

To determine the expression profile of *sad-8*, we examined its transcript levels using RNA-seq datasets (see *Materials and Methods*). Most MSUD genes that are not known to affect quelling (vegetative silencing), such as *sad-1* to *sad-5*, have relatively low vegetative expression (as compared to their sexual expression) (Decker *et al.* 2017). *sad-8*, on the other hand, has robust expression during vegetative growth, suggesting that it may have a role in the asexual stage (Table 3). Unsurprisingly, *sad-8* is well expressed during the sexual cycle.

**Table 3:** Expression profiles of silencing genes

Gene Name	Gene No.	Vegetative Expression (FPKM)	Sexual Expression (FPKM)
Housekeeping			
* actin*	*ncu04173*	2638.3425	905.4051
MSUD			
* sad-5*	*ncu06147*	0.0000	13.2559
* sad-6*	*ncu06190*	12.1721	20.5948
* sad-7*	*ncu01917*	0.6982	12.5427
* sad-8*	*ncu01310*	67.4554	66.8362
MSUD/Quelling			
* qip*	*ncu00076*	18.6841	107.2514

Quelling is the vegetative RNA silencing system that targets tandem transgenes in *Neurospora*. FPKM, fragments per kilobase of exon per million mapped reads.

### SAD-8 is not required for either vegetative growth or sexual development

**Figure 3:**
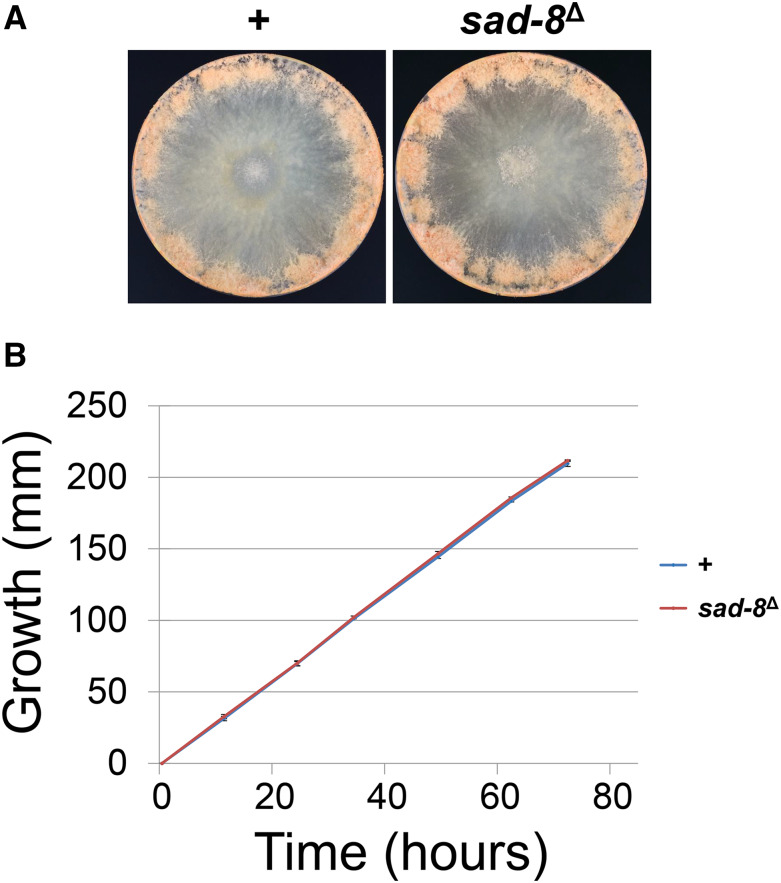
SAD-8 is not essential for the vegetative cycle. Deletion of *sad-8* does not have a substantial effect on either conidiation (A) or linear growth (B). Growth assays were performed in triplicate. +, wild type (WT) at the *sad-8* locus. Strains used in this study: P3-08 and P23-11.

Thus far, none of the known MSUD proteins are essential for cell viability (Samarajeewa *et al.* 2017). As demonstrated in Figure 3, if *sad-8* is functional during the asexual stage (a speculation based on its expression profile), it is unlikely to be a crucial factor in growth and development, as the corresponding deletion strain is proficient in linear growth and conidial production.

**Figure 4:**
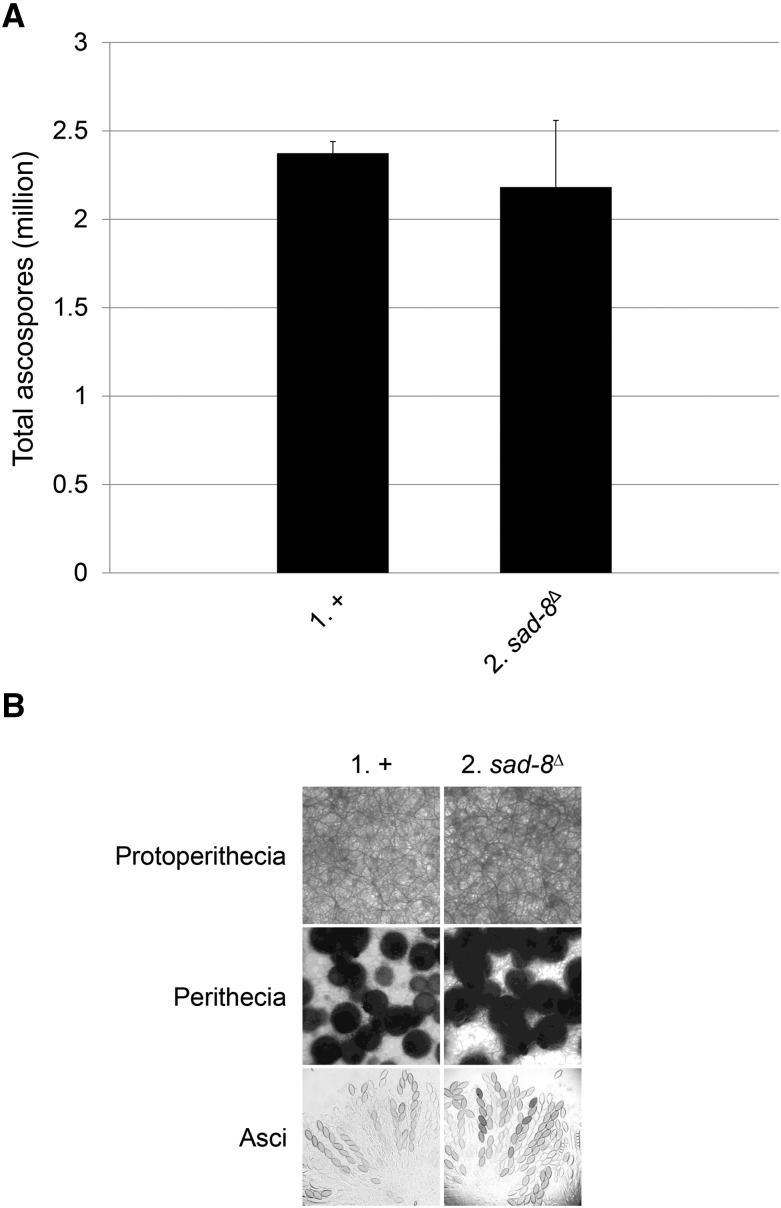
SAD-8 is not essential for the sexual cycle. (A) Ascospore production appears proficient in a *sad-8*-null cross. (B) Deletion of *sad-8* does not substantially affect the development of protoperithecia, perithecia, and/or asci. 1, F2-01 × P3-08. 2, F7-14 × P23-11.

On the other hand, there is a history of MSUD players being important for the sexual stage. For example, crosses lacking *dcl-1* or *qip* produce perithecia devoid of asci, suggesting that the two genes are necessary for early sexual development (Alexander *et al.* 2008; Xiao *et al.* 2010). Crosses lacking *sad-1*, *sad-2*, or *sad-3* have a less dramatic phenotype, as they make asci that abort before ascospore formation (Shiu *et al.* 2001, 2006; Hammond *et al.* 2011a). Hence, it was once thought that sexual development may require some degree of meiotic silencing. This notion was later rejected with the discovery of *sad-4*, *sad-5*, and *sad-6*, as a cross missing any one of these genes can still produce an appreciable number of ascospores (Hammond *et al.* 2013b; Samarajeewa *et al.* 2014). As for *sad-8*, its deletion does not materially affect the development of protoperithecia, perithecia, asci, and/or ascospores, suggesting that this gene is not essential for the sexual stage (Figure 4).

### SAD-8 is an essential component of MSUD

**Figure 5:**
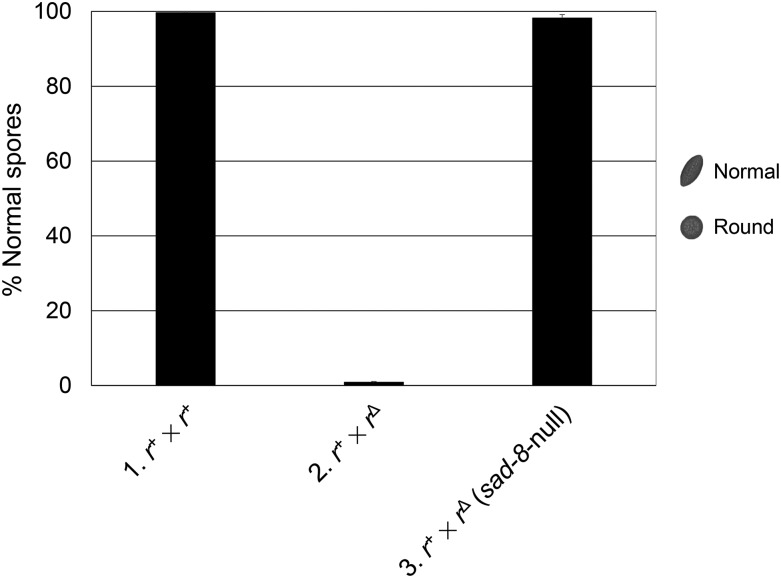
The loss of SAD-8 leads to a near-complete deficiency in MSUD activity. (A) In a normal cross, most ascospores are of American football shape (99.7%). (B) If the *r*^+^ gene is unpaired in an MSUD-proficient cross, most progeny are round (*i.e.*, 0.9% football). (C) The silencing of an unpaired *r*^+^ is suppressed in a *sad-8*-null background (*i.e.*, 98.3% football). Crosses were performed in triplicate. 1, F2-01 × P3-08. 2, F2-29 × P3-08. 3, F7-14 × P23-12.

For an MSUD gene that is necessary for sexual development, it is difficult to evaluate its true impact on silencing because we can at most knock down its expression (via the “silencing the silencer” scheme). However, since *sad-8* is dispensable for ascus and ascospore maturation, we can determine whether it plays a critical or auxiliary role in MSUD. In a cross homozygous for *sad-8*^∆^, the silencing of an unpaired *r*^+^ seems to be almost completely deficient, suggesting that this *sad* gene is essential for MSUD (Figure 5).

### Localization of SAD-8

**Figure 6:**
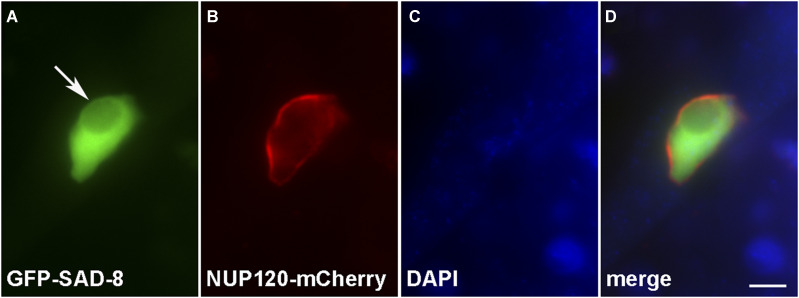
SAD-8 localizes predominantly in the nucleus. SAD-8 is found mainly in the nucleus, excluding the nucleolus (arrow). Micrographs illustrate prophase asci expressing *gfp-sad-8* and *nup120-mCherry* (P27-13 × P27-14). The nuclear envelope was marked by nucleoporin NUP120. The chromatin was stained with DAPI. Bar, 5 μm.

The first few reported MSUD proteins localize in the perinuclear region, the epicenter of meiotic silencing activity (Shiu *et al.* 2006; Alexander *et al.* 2008; Xiao *et al.* 2010; Hammond *et al.* 2011a, 2013b). These proteins form an RNAi complex (MSC), which searches for and modifies certain exported RNAs (Decker *et al.* 2015). The discovery of SAD-5 and SAD-6 reaffirmed a long-held belief that some parts of the silencing machinery must locate inside the nucleus (Hammond *et al.* 2013b; Samarajeewa *et al.* 2014). Since SAD-8 has similarity with a human nuclear cap-binding protein (NCBP3), it stands to reason that it may also be found mainly in the nucleus. To test this theory, we tagged SAD-8 with the green fluorescent protein (GFP) and examined its subcellular localization. Not surprisingly, SAD-8 appears to be predominantly nuclear (Figure 6), much like both CBC subunits (Decker *et al.* 2017).

### Interaction between SAD-8 and CBC

**Figure 7:**
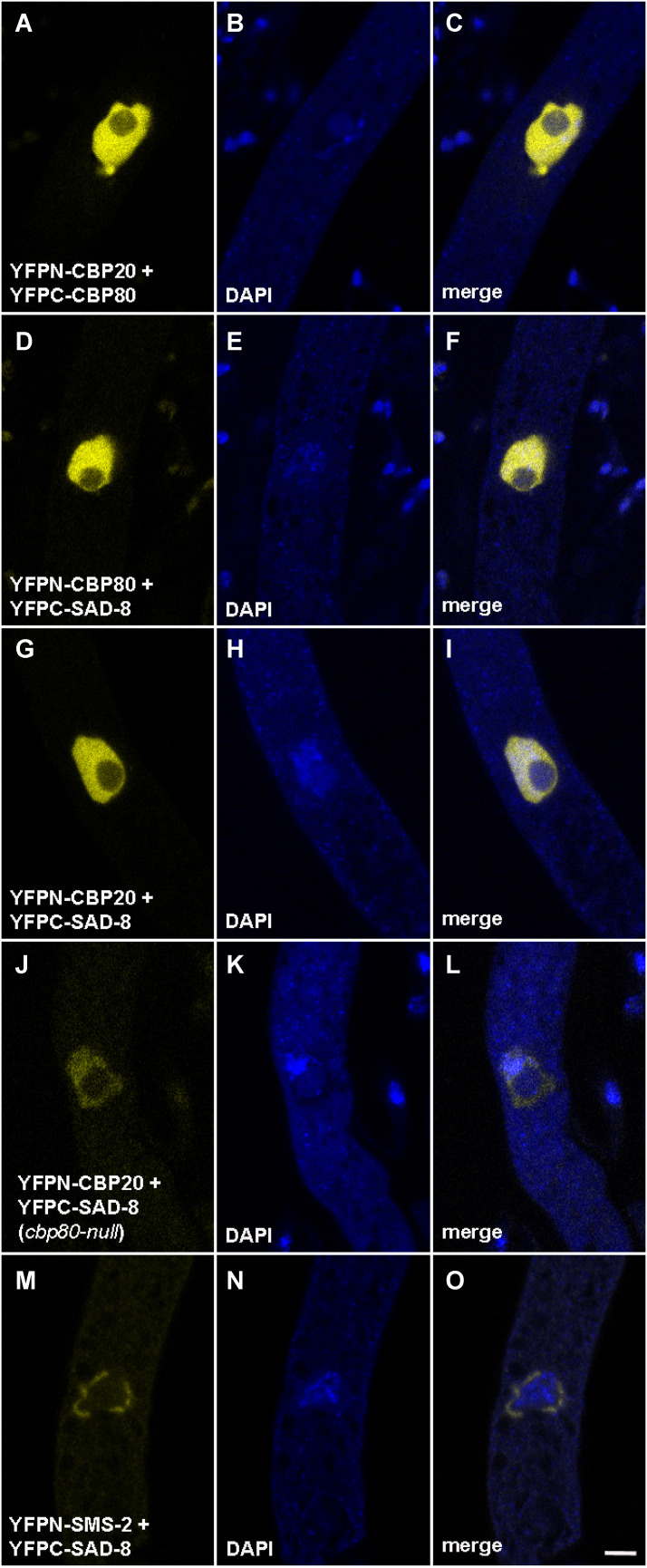
Interactions among CBP20, CBP80, SAD-8, and SMS-2. In a BiFC assay, a positive interaction reconstitutes the yellow fluorophore. CBP80 (NCBP1) and CBP20 (NCBP2) form the heterodimeric CBC (C). SAD-8 (NCBP3 homolog) has interaction with both components of CBC (F and I) and the SMS-2 Argonaute (O). CBP20 interacts with SAD-8 even in the absence of CBP80 (L). Micrographs illustrate prophase asci expressing (A–C) *yfpn-cbp20* and *yfpc-cbp80* (P21-10 × P21-11), (D–F) *yfpn-cbp80* and *yfpc-sad-8* (P27-15 × P27-16), (G–I) *yfpn-cbp20* and *yfpc-sad-8* (P25-68 × P25-69), (J–L) *yfpn-cbp20* and *yfpc-sad-8* in a *cbp80*-null background (F8-36 × P27-17), and (M–O) *yfpn-sms-2* and *yfpc-sad-8* (P25-72 × P26-01). Bar, 5 μm.

In mammals, NCBP1 (CBP80) forms a complex (canonical CBC) with NCBP2 (CBP20) and another complex (alternative CBC) with NCBP3 (SAD-8 homolog) (Gebhardt *et al.* 2015). To determine if a similar complex formation pattern is present in *Neurospora*, we utilized BiFC to investigate the interactions among the corresponding proteins. As expected, *Neurospora* CBP80 interacts with both CBP20 and SAD-8 (Decker *et al.* 2017; Figure 7, C and F).

In humans, NCBP2 and NCBP3 use different domains (RRM and C-terminal region, respectively) to interact with NCBP1 (Gebhardt *et al.* 2015). This may suggest that NCBP1 also uses different domains to interact with NCBP2 and NCBP3, leaving the possibility of an NCBP1/2/3 trimerization. While Gebhardt *et al.* (2015) did not present a model involving the complex formation of CBC-NCBP3, such association has been shown in a co-elution study using heterologously expressed human proteins (Schulze *et al.* 2018). We asked if this interaction can also be seen *in vivo* in a nonmammalian system. Indeed, CBP20 and SAD-8 have physical association in *Neurospora* (Figure 7I), suggesting that the CBC-NCBP3 interaction reported by Schulze *et al.* (2018) is not an anomaly. A simple explanation for our observations is that CBP20, CBP80, and SAD-8 form a complex in *Neurospora*.

It is possible that CBP20 and SAD-8 do not have direct interaction and that they are close to one another simply because they both have an affinity for CBP80. As shown in Figure 7L, the interaction between CBP20 and SAD-8 can still be detected in a *cbp80*-null background, demonstrating that the two proteins can in fact come together independently of CBP80. This finding further supports that CBC and SAD-8 have the ability to assemble into a complex.

### SAD-8 interacts with an MSC component

CBC travels in and out of the nucleus to perform its miscellaneous functions (Gonatopoulos-Pournatzis and Cowling 2014). In *Neurospora*, it interacts with the SMS-2 Argonaute (an MSC component) and plays a role in MSUD (Decker *et al.* 2017). We asked if SAD-8, an NCBP3-like protein that interacts with CBC, also has physical association with SMS-2. Indeed, SAD-8 interacts with SMS-2 in the perinuclear region (Figure 7O). This suggests that SAD-8, like CBC, mediates silencing through its interaction with MSC.

## Discussion

In *Neurospora*, cross walls between adjacent cells are normally incomplete, and selfish DNA elements can potentially permeate the entire fungal network. Accordingly, genome defense mechanisms such as repeat-induced point mutation (Cambareri *et al.* 1989), quelling (Romano and Macino 1992), and MSUD (Shiu *et al.* 2001) are preserved in this fungus. MSUD, for example, can keep transposons in check during the sexual stage (Wang *et al.* 2015). We have previously shown that CBC, which plays a role in various post-transcriptional processing events, is involved in MSUD (Decker *et al.* 2017). This study identifies a CBC-linked factor (SAD-8) as a major player of MSUD, further connecting cap-binding proteins to silencing.

SAD-8 is similar to NCBP3, which binds NCBP1 and forms an alternative CBC in mammals (Gebhardt *et al.* 2015, 2019). While the canonical and alternative CBCs are somewhat redundant in their functions, the latter plays a primary role in promoting appropriate gene expression during environmental challenges. For example, NCBP3 is important for cytokine mRNA translation and proper antiviral immune responses. NCBP3 is not as ubiquitous as NCBP1 and NCBP2; NCBP3-family proteins are not found in plants and some popular animal and fungal models (*e.g.*, *Drosophila melanogaster* and *Saccharomyces cerevisiae*). In Pezizomycotina, the subphylum to which *Neurospora* belongs, five classes do not have an NCBP3 homolog (Figure 2). Gebhardt *et al.* (2015) suggested that the loss of an NCBP3 protein may be a result of evolutionary adaptation. For example, while *Homo sapiens* may keep an NCBP3 protein for spliced mRNA biogenesis and *N. crassa* may keep another for silencing, *S. cerevisiae* may not need any as it possesses very few introns and has lost its ability to conduct RNAi (Ast 2004; Drinnenberg *et al.* 2009). It remains to be seen whether an NCBP3-family protein is required for silencing in other organisms, although both *Neurospora tetrasperma* and *Fusarium graminearum*, two fungi that are MSUD-capable (Ramakrishnan *et al.* 2011; Son *et al.* 2011), contain a SAD-8 homolog (data not shown).

Unlike the first few reported MSUD components, SAD-8 joins a growing list of silencing factors that are not absolutely required for sexual spore production. Deletion of *sad-8* does not appear to affect either somatic growth or asexual spore production under standard laboratory conditions, although its expression profile suggests that it may play a role in the asexual stage. In HeLa cells, NCBP3 depletion causes an almost 100-fold increase in viral growth (Gebhardt *et al.* 2015). In mice, NCBP3- deficient individuals have severe lung pathology and increased mortality after influenza A virus infection (Gebhardt et al. 2019). It is possible that a *sad-8* mutation may show an adverse effect under stressful conditions.

Since *sad-8* is not required for sexual development, it is possible to fully assess its silencing role in a homozygous deletion cross. Interestingly, while a cross devoid of CBC still maintains roughly four-fifths of its MSUD activity (Decker *et al.* 2017), the absence of *sad-8* leads to a near-complete silencing deficiency. These suggest that while CBC plays an auxiliary role in MSUD, SAD-8 is a critical silencing component.

Like other RNA export proteins, human NCBP3 is predominantly nuclear but can also be found in the cytoplasm (Gebhardt *et al.* 2015). In *Neurospora* asci, SAD-8 behaves similarly as it localizes predominantly in the nucleus but can also be seen in the perinuclear region (Figure 7O). In mammals, NCBP3 is hypothesized to form an alternative CBC with NCBP1 (Gebhardt *et al.* 2015, 2019). However, Schulze *et al.* (2018) showed that it is possible to reconstitute a CBC-NCBP3 complex *in vitro* and proposed a model for its biological relevance. While other models of complex assembly are possible, a straightforward interpretation of our results is that CBP20, CBP80, and SAD-8 form a trimer in *Neurospora*. Like CBC, SAD-8 has interaction with the SMS-2 Argonaute, a component of MSC. This observation solidifies the connection of *Neurospora* nuclear cap-binding proteins to RNAi and supports the notion that they mediate MSUD through their interactions with the perinuclear silencing complex.

According to our MSUD model, an unpaired gene initiates the production of aRNAs, which are exported to the perinuclear region and processed into siRNAs. These siRNAs can then guide MSC to target complementary mRNA transcripts. An mRNA is typically bound by CBC, which mediates its export to the cytoplasm. One possibility is that the CBC-SAD-8 complex helps deliver an exiting mRNA to MSC. In this scenario, the contact between SAD-8 and MSC is essential for the mRNA transfer, and CBC enhances their interaction. In the absence of CBC, a SAD-8-bound mRNA can still be exported to the perinuclear region and interact with MSC, even though it does not have the optimal configuration for the silencing machinery. While this is a conceivable explanation for our observations, other possibilities abound. For example, CBC and/or SAD-8 may have a stimulatory effect on the silencing machinery directly or indirectly (Sabin *et al.* 2009). Future studies on these cap-binding proteins should give us more insights into how they are involved in the MSUD pathway.
